# The Ketogenic Diet and Cardiovascular Diseases

**DOI:** 10.3390/nu15153368

**Published:** 2023-07-28

**Authors:** Damian Dyńka, Katarzyna Kowalcze, Anna Charuta, Agnieszka Paziewska

**Affiliations:** Institute of Health Sciences, Faculty of Medical and Health Sciences, Siedlce University of Natural Sciences and Humanities, 08-110 Siedlce, Poland; damian.dynka24@gmail.com (D.D.); katarzyna.kowalcze@uph.edu.pl (K.K.); anna.charuta@uph.edu.pl (A.C.)

**Keywords:** ketogenic diet, cardiovascular disease, prevention, treatment, KD, CVD, heart, ketone bodies, ketone, diet, inflammation, endothelium, lipid profile, cholesterol, LDL, HDL, triglyceride, low carb, high fat, fatty acids, heart metabolism, cardiomyocytes, blood pressure, diastolic, systolic, obesity, weight loss, reduction

## Abstract

The most common and increasing causes of death worldwide are cardiovascular diseases (CVD). Taking into account the fact that diet is a key factor, it is worth exploring this aspect of CVD prevention and therapy. The aim of this article is to assess the potential of the ketogenic diet in the prevention and treatment of CVD. The article is a comprehensive, meticulous analysis of the literature in this area, taking into account the most recent studies currently available. The ketogenic diet has been shown to have a multifaceted effect on the prevention and treatment of CVD. Among other aspects, it has a beneficial effect on the blood lipid profile, even compared to other diets. It shows strong anti-inflammatory and cardioprotective potential, which is due, among other factors, to the anti-inflammatory properties of the state of ketosis, the elimination of simple sugars, the restriction of total carbohydrates and the supply of omega-3 fatty acids. In addition, ketone bodies provide “rescue fuel” for the diseased heart by affecting its metabolism. They also have a beneficial effect on the function of the vascular endothelium, including improving its function and inhibiting premature ageing. The ketogenic diet has a beneficial effect on blood pressure and other CVD risk factors through, among other aspects, weight loss. The evidence cited is often superior to that for standard diets, making it likely that the ketogenic diet shows advantages over other dietary models in the prevention and treatment of cardiovascular diseases. There is a legitimate need for further research in this area.

## 1. Introduction

Cardiovascular diseases (CVD) are the most common global cause of death. Each year, an average of 17.9 million people worldwide die from them [1]. In 2020, the number of deaths due to CVD was over 19 million, a significant increase of 18.7 per cent from 2010 [2]. The statistics are even worse when comparing 1990 with 2019, with CVD deaths rising from 12.1 million to 18.6 million in this period [3]. Thus, they represent a significant and increasing concern, even compared to cancer (approximately 10 million deaths per year) [4]. Significant increases in CVD incidence and deaths were predicted many years ago. The authors of a 2006 publication predicted around 23.3 million CVD deaths by 2030 [5]. Unfortunately, these predictions appear to be increasingly realistic, despite significant medical developments in recent years.

Cardiovascular diseases include coronary heart disease, heart failure, cardiac arrhythmia, cerebrovascular disease, valvular heart disease, pericardial disease, cardiomyopathy (heart muscle disease), congenital heart defects, rheumatic heart disease, sudden cardiac arrest, atherosclerosis, heart attacks, strokes, dyslipidemia, hypertension and others [2]. A poor diet, a lack of or low physical activity, smoking, alcohol consumption, obesity, diabetes, chronic stress and vitamin D deficiency are considered to be some of the main causes of the development of heart disease. Hypertension is a disease in itself, but, indirectly, it significantly increases the risk of developing further cardiovascular diseases. High blood pressure is observed in as many as seven out of ten people with a first heart attack and eight out of ten people with a first stroke. In addition, excessive sodium intake and excessively high serum LDL lipoprotein levels are also identified as risk factors [1,6,7,8]. Although some risk factors are not firmly established in the scientific evidence, they are still considered to be among the main ones. Meanwhile, others (including homocysteine) are often overlooked, whereas they may often be more meaningful indicators [9,10,11]. Taking all these factors into account, it seems that diet plays a key role. It is in itself an important element in the prevention and treatment of CVD and, on the other hand, influences indirect risk factors [12,13]. The standard recommended dietary management for the prevention and treatment of cardiovascular diseases includes high intake of vegetables, fruit, fish, legumes and whole grain products [14]. This is in line with the structure (specificity) of the Mediterranean and DASH diets, among others [15,16,17,18].

A remarkably different approach in the prevention and treatment of cardiovascular disease is the ketogenic diet, which places the body into a state of ketosis. In this case, the main source of energy for the body is ketone bodies, produced from fats. This distinguishes this diet from all others, in which the main source of energy is glucose. In order to achieve a state of nutritional ketosis, the proportion of energy coming from fat in such a diet is usually 70–80% (up to 90% in the strictly clinical version used, for example, in the treatment of epilepsy), with protein around 20%, and carbohydrates rarely exceed 50 g per day [19]. Due, among other aspects, to the poor reputation of fats in terms of the risk of developing cardiovascular diseases, there is much controversy about the impact of this diet on these diseases. According to the American Heart Association (AHA), the ketogenic diet also raises some controversy related to CVD and has poor alignment with the AHA dietary guidelines [20]. However, recent scientific evidence suggests that it may be a promising direction for further research. The following sections of this article will discuss, in the context of the latest scientific evidence, the many inaccuracies and uncertainties associated with the use of the ketogenic diet model.

## 2. The Ketogenic Diet and Blood Lipid Profile

### 2.1. Lipid Profile and Cardiovascular Diseases

The blood lipid profile is most commonly studied in the form of four parameters, i.e., total cholesterol (TC), low-density lipoprotein (LDL), high-density lipoprotein (HDL) and triglycerides (TG). Abnormalities in these parameters (dyslipidemia) are considered one of the main risk factors for cardiovascular diseases [21].

The World Health Organization lists high total cholesterol as one of the risk factors for these diseases [1]. A 2022 publication summarizing selected studies in this area found a relationship between higher TC levels and increased cardiovascular mortality [22]. Another prospective 10-year cohort study showed the highest risk of ischemic heart disease at total cholesterol levels <155 mg/dL and >185 mg/dL [23]. However, there are large discrepancies between the results of different studies due to the different methodological selections. A prospective cohort study of 12.8 million adults found that the lowest mortality from any cause was associated with total cholesterol levels of 210–249 mg/dL. This was true for every age–gender group except men and women aged 18–34 years (180–219 mg/dL for men and 160–199 mg/dL for women) and women aged 35–44 years (180–219 mg/dL) [24]. Similar results were presented by the British Heart Foundation and the World Health Organization as early as 2005, based on data from 164 countries from around the world. They showed that the lowest mortality from any cause was associated with TC concentrations between 200 mg/dL and 240 mg/dL. The lowest mortality from cardiovascular diseases was presented in the range of approximately 190–225 mg/dL [25]. The authors of another publication raised the issue of serious methodological errors (including ignoring contradictory information or using misleading statistics) in studies suggesting higher TC and LDL concentrations as a major cause of CVD [26].

Low-density lipoprotein (LDL) is considered the so-called “bad cholesterol” and an even more significant risk factor than TC [27,28,29]. A 2023 publication showed that the effect of the LDL cholesterol concentration on the risk of sudden cardiac arrest (SCA) followed a “U” shape, i.e., the highest risk occurred at the lowest and highest LDL concentrations [30]. It further appears that the lowest and highest LDL concentrations are also associated with the overall risk of cardiovascular death. However, it is worth noting that very low LDL concentrations are additionally strongly associated with the risk of death from any cause [31]. However, given the different methodological approaches and the suggestions of the authors of another publication, there is no strong evidence for the causality of LDL in the development of CVD [26]. It is therefore worth noting that a correlation does not imply causality. In addition, the size of the LDL particle itself may be relevant, as it appears that smaller LDL particles are potentially more detrimental compared to larger ones [32].

High-density lipoprotein (HDL) is considered the so-called “good cholesterol” and, contrary to LDL, an increase in its concentration is considered a beneficial effect [33]. Publications show that it is inversely correlated with CVD mortality [22], although both too low and too high concentrations of it may be unfavorable, according to some studies [34,35].

High triglyceride levels are also considered to increase CVD risk [36]. A 10-year-long cohort study from 2021 showed that there was a significant, linear association of triglyceride concentrations with ischemic stroke. However, it appears that here, too, excessively low concentrations (<80 mg/dL) may be associated with an increased risk of death from any cause [23]. There is even the so-called triglyceride paradox, as triglyceride concentrations are inversely correlated with mortality from any cause, even among patients with CVD [37,38]. The TG/HDL ratio itself may be much more meaningful than the TG concentration in predicting CVD risk [39].

A 2023 publication showed that residual cholesterol is an independent (and possibly earlier than LDL) risk factor for atherosclerosis [40]. Another study showed that both residual cholesterol and LDL cholesterol were equally associated with the risk of ischemic heart disease. Despite this, only residual (non-fasting) cholesterol levels were associated with an increased risk of death from any cause [41]. Another publication showed that the ratio of apolipoprotein B (Apo B) to apolipoprotein AI (Apo A-I) was a much better predictor in assessing cardiovascular risk. At the same time, the significance of other parameters, such as TC, LDL, TG and TC/HDL-c, TG/HDL-c and LDL-c/HDL-c ratios, was not demonstrated [42].

### 2.2. The Effect of the Ketogenic Diet on the Blood Lipid Profile

The impact of the ketogenic diet on the blood lipid profile is undoubtedly controversial. There are many conflicting data, which may be due to a misunderstanding of certain facts and research methodologies. The high-fat (with frequent high cholesterol) nature of this dietary model contributes to this. As is well known, there is a great deal of controversy about the impact of the amount and type of fat and cholesterol consumed on the blood lipid profile.

For a detailed and thorough analysis of the effect of the ketogenic diet on the lipid profile, it is worth discussing recent randomized controlled trials (RCTs) and contrasting them with previous publications on the topic. The authors of a 2022 RCT compared the effect of the ketogenic diet (KD) vs. the standard diabetes diet (SDD) in overweight or obese patients recently diagnosed with type 2 diabetes. Considering the lipid profile, it appeared that the ketogenic diet showed a greater benefit. There was a greater reduction in total cholesterol (from 4.54 ± 0.69 mmol/L to 4.02 ± 0.43 mmol/L vs. from 4.56 ± 0.67 mmol/L to 4.23 ± 0.47 mmol/L in those in the SDD group), triglycerides (from 1.76 ± 0.59 mmol/L to 1. 44 ± 0.26 mmol/L vs. from 1.81 ± 0.78 mmol/L to 1.66 ± 0.46 mmol/L in SDD subjects) and LDL fraction cholesterol (from 2.75 ± 0.65 mmol/L to 2.34 ± 0.45 mmol/L vs. from 2.77 ± 0.69 mmol/L to 2.59 ± 0.58 mmol/L in SD subjects). In addition, HDL fraction cholesterol changed more favorably (increasing from 1.08 ± 0.11 mmol/L to 1.21 ± 0.23 mmol/L vs. from 1.09 ± 0.19 mmol/L to 1.12 ± 0.20 mmol/L in those in the SDD group) [43]. It is important that despite the assumption of the same calorie consumption in both groups (1500 ± 50 kcal), the body weights of participants using KD decreased on average from 78.32 ± 15.27 to 70.26 ± 14.79 over 12 weeks. In comparison, the body weights of participants using SDD did not change (from an average of 77.95 ± 14.76 kg to 77.34 ± 13.28 kg). On the one hand, this is an argument in favor of the ketogenic diet; on the other hand, it should be noted that greater benefits in the form of changes in the lipid profile may also result directly from weight loss among participants using KD. In addition, it could also have been influenced by the composition of the diet itself, in which there was a significant share of unsaturated fatty acids from olive oil, avocado and fish.

Another RCT from 2022 compared the effect of the well-formulated ketogenic diet (WFKD) with the Mediterranean-plus diet (Med-Plus) among patients with diabetes and pre-diabetes. The advantage of the ketogenic diet was evidenced by a greater reduction in triglycerides from 118.8 mg/dL to 99.5 mg/dL (in Med-Plus from 131.1 mg/dL to 121.7 mg/dL) and a greater increase in HDL fraction cholesterol from 49.1 mg/dL to 54.1 mg/dL (in Med-Plus from 48 mg/dL to 47.9 mg/dL). However, the study showed an increase in the LDL cholesterol fraction (from 97.8 mg/dL to 111.3 mg/dL) among those on the ketogenic diet, while there was a reduction among the Med-Plus group (from 111.5 mg/dL to 95.3 mg/dL) [44]. Another RCT was conducted in 2022, which, in turn, assessed, among other factors, the effect of the ketogenic diet on the lipid profile among patients with severe obstructive sleep apnea syndrome. The diet had a beneficial effect on the lipid profiles of the patients, which was observed as, among other factors, a reduction in total cholesterol from 200.1 ± 30.1 mg/dL to 180.4 ± 35.2 mg/dL, LDL from 127.4 ± 26.8 mg/dL to 107.1 ± 37.1 mg/dL and triglycerides from 191 ± 41.7 mg/dL to 130 ± 79 mg/dL. At the same time, it is worth mentioning that these results were due to the simultaneous use of the low-calorie ketogenic diet (LCKD) with continuous positive airway pressure (CPAP). They were superior to those of an intervention limited to CPAP alone. HDL fraction cholesterol was not significantly altered in either group [45]. However, the observed greater benefits in the CPAP + LCKD group may have resulted from the much greater weight loss (on average from 143.6 ± 23.6 kg to 129.7 ± 23.7 kg vs. from an average of 132.7 ± 23 kg to 131.6 ± 22.3 kg in the CPAP group).

Another randomized controlled trial among trained men, on the other hand, showed no significant differences in lipid profile changes between ketogenic (KD) and non-ketogenic (NKD) diets [46]. The authors of another RCT further showed that the ketogenic diet increased LDL cholesterol levels in every healthy young woman of normal weight, with a treatment effect of 1.82 mM (*p* < 0.001). The diet also increased the concentrations of small dense LDL cholesterol and large floating LDL cholesterol. In addition, an increase in apolipoprotein B-100 (ApoB) was observed. The authors, therefore, concluded that the ketogenic diet in this case contributed to the worsening of the blood lipid profile in the women studied [47]. It is worth noting that this is one of the studies in which there was no weight loss, significantly improving the lipid profile in the body.

On the other hand, another publication responding to these results suggests that this is not a cause for concern. The author argues that although many studies show an association between LDL-C and cardiovascular diseases, this is not conclusive of a causal relationship [48]. Another randomized controlled trial, however, reported a beneficial effect of the ketogenic diet on the lipid profile, including a reduction in LDL fraction cholesterol. Importantly, benefits were observed regardless of the type of protein consumed—in the groups consuming whey protein, animal protein and vegetable protein. In addition to a significant reduction in LDL, there was a significant reduction in total cholesterol and triglycerides. However, the HDL fraction did not change much [49]. Another RCT conducted among women with hyperinsulinemia and excessive body weight, on the other hand, showed that HDL concentrations increased significantly in women following a low-calorie ketogenic diet (LCKD), from 36.71 ± 4.42 mg/dL to 52.99 ± 7.77 mg/dL. This was significantly better than the control group (CG), in which there was no significant change in HDL levels (from 44.14 ± 5.07 to 43.01 ± 5.03 mg/dL). Furthermore, triglycerides (TG) in LCKD decreased from 213.45 ± 63.60 mg/dL to 129.13 ± 46.23 mg/dL, compared to the control group from 210.57 ± 36.45 mg/dL to 206.44 ± 50.03 mg/dL [50]. The observed changes may have been mainly due to weight loss alone (from 89.08 ± 14.68 kg to 75.36 ± 13.47 kg vs. 90.63 ± 11.04 kg to 89.86 ± 11.30 kg in CG) and a reduction in waist circumference among the LCKD group (from 101.04 ± 11.86 cm to 87.34 ± 9.50 cm vs. from 102.93 ± 10.32 cm to 103.67 ± 9.79 cm in CG). This was most likely due to the fact that the two groups were not equivalent in terms of calorie intake.

Given the inconclusive results, it is worth citing a longer study. Saslow et al. reported the results of a 12-month-long randomized trial of a moderate- to very low-carbohydrate diet in overweight adults with type 2 diabetes or pre-diabetes. The results confirmed the benefits of the ketogenic diet on triglyceride and HDL fraction cholesterol levels both after six months and a full year. However, it is noteworthy that the LDL cholesterol fraction values were increased during this time [51] so the results were also not clear.

Detailed results are included in Table 1. There is also an RCT that examined the effect of omega-3 fatty acid supplementation in the Mediterranean ketogenic diet on some cardiovascular risk factors. Importantly, both the Mediterranean ketogenic diet alone and that enriched with omega-3 fatty acid supplementation were able to lower total cholesterol, triglycerides and LDL fraction cholesterol and increase HDL fraction cholesterol [52]. The authors of another RCT also studied the effect of a ketogenic diet in obese children and adolescents. It was shown that the ketogenic diet increased HDL fraction cholesterol concentrations to some extent and lowered triglyceride concentrations. However, at the same time, it also increased total cholesterol and LDL fraction cholesterol concentrations, which indicates mixed results [53]. All the randomized controlled trials described are illustrated in Table 1.

Heterogeneous results in randomized controlled trials translate directly into a lack of unified consensus in published meta-analyses and systematic reviews. In one meta-analysis, the authors compared the benefits of a ketogenic diet and conventional non-ketogenic diets in cancer patients. One of the indicators studied was the lipid profile. The ketogenic diet was shown to lower triglycerides (WMD = −24.46 mg/dL; 95% CI: −43.96; −4.95; and *p* = 0.014) and showed an insignificant beneficial effect on cholesterol [54]. In another meta-analysis, the authors concluded that patients with type 2 diabetes on a ketogenic diet were not associated with increased levels of total cholesterol or LDL fraction or reduced levels of HDL fraction. In addition, compared to the control group, triglyceride concentrations decreased after the ketogenic diet (SMD = −0.49, 95% CI: −0.82 to −0.17, *p* = 0.003) [55]. The results of another meta-analysis in 2022 indicated that the ketogenic diet, even over 12 months, was more effective (compared to the control group) in lowering triglycerides and increasing HDL fraction cholesterol [56]. Yuan et al. conducted a meta-analysis comparing the parameters of patients with type 2 diabetes before and after the ketogenic diet. Triglycerides were shown to decrease by an average of 0.72 (95% CI: −1.01 to −0.43), total cholesterol by 0.33 (95% CI: −0.66 to −0.01) and LDL by 0.05 (CI: −0.25 to −0.15), after KD treatment while HDL concentrations increased by 0.14 (95% CI: 0.03 to 0.25). In view of this, the authors of the meta-analysis concluded that KD had a beneficial effect in improving lipid parameters in patients with T2DM [57]. The results of a meta-analysis by López-Espinoza et al. showed no greater benefits of a ketogenic diet (KD) compared to a balanced diet among obese patients. At the same time, this provides evidence that KD is not characterized by worse outcomes compared to a balanced diet [58]. In contrast, another meta-analysis evaluating the potential of the very low-calorie ketogenic diet (VLCKD) in the treatment of obesity showed its superiority over other diets in improving a number of parameters, including total cholesterol and triglycerides. Significant reductions in LDL cholesterol, among others, were also observed, but these were similar to changes with other weight loss interventions [59]. The efficacy and safety of VLCKD in overweight and obese patients was also evaluated by Castellana et al. The authors indicated that with VLCKD, there was a mean reduction in total cholesterol (−28 mg/dL) and triglycerides (−30 mg/dL), while there were no significant changes in HDL and LDL cholesterol fractions [60].

Often, the high cholesterol intake of the ketogenic diet is cited as a cause of increased serum cholesterol levels. Meanwhile, there is no strong, unequivocal evidence to suggest that there is a risk of an increase in serum cholesterol due to an increased supply of cholesterol from food. As a result of regulatory mechanisms, the body is able to take in as much cholesterol as it needs. In fact, even the consumption of 25 eggs (which are known to be rich in cholesterol) per day for 15 years did not result in an abnormal lipid profile in an 88-year-old patient [61]. Furthermore, the upper limit on dietary cholesterol intake was eliminated in 2015 from the United States Department of Agriculture (USDA) recommendations. Recent publications confirm that there is no direct relationship between dietary cholesterol intake and serum cholesterol levels [62].

Given the totality of the evidence of the effect of the ketogenic diet on the blood lipid profile, there is certainly a preponderance of evidence of a beneficial effect. There is little evidence to suggest that the ketogenic diet has a worse effect on the overall lipid profile compared to other diets. Often, even if it does not show any greater benefits, it has a comparably beneficial effect to the control diet. This is all the more optimistic given that studies suggesting the cholesterol hypothesis in the context of cardiovascular risk do not meet the Bradford Hill criteria for causality [26]. However, the results of the studies are inconclusive and this should be noted. In many studies, there is a reduction in the LDL cholesterol fraction. On the other hand, however, even when it is increased in other studies, the correlation of LDL with cardiovascular diseases does not necessarily imply causality. It has even been shown that people with the highest LDL fraction cholesterol concentrations live as long as or, in most cases, longer than those with normal or low LDL concentrations [63,64]. Nevertheless, no clear consensus can be formed on the basis of the available research results, although the available scientific evidence mostly shows a positive effect of the ketogenic diet on the blood lipid profile.

## 3. Anti-Inflammatory Potential of the Ketogenic Diet in Cardiovascular Diseases

An indispensable element in the development of cardiovascular diseases is the involvement of inflammation, as demonstrated in a number of studies [65,66,67,68,69,70]. Inflammation has been shown to initiate the early stages of the atherosclerotic process. In addition, as pro-inflammatory cytokines increase, the risk of developing CVD increases. It appears that innate immunity plays a key preventive role here [65,71]. However, excessive inflammation influences endothelial dysfunction and, as a result, a number of processes (including increased lipoprotein permeability) that result in far-reaching consequences related to, among other outcomes, the development of atherosclerosis [72]. Furthermore, psychological stress also initiates inflammation, contributing to an increased risk of developing CVD. This is mediated by an increase in sympathetic nervous system activity as a result of a number of changes occurring related, inter alia, to an increase in the metabolic activity of specific brain areas [73,74,75,76,77]. This is well supported by the fact that the increased metabolic activity of these areas alone makes it possible to predict the development of coronary heart disease, irrespective of standard risk factors for this disease [78]. Importantly, the ageing process itself is also associated with low-grade inflammation, which consequently increases the risk of developing an atherosclerotic process [72].

**Figure 1:**
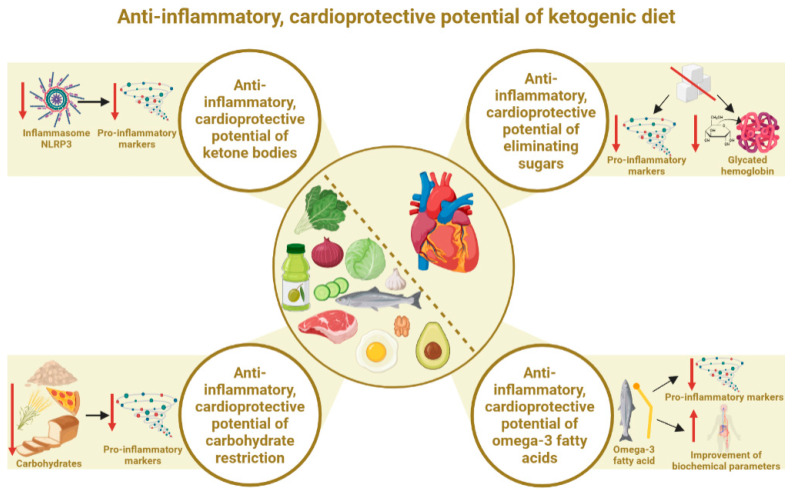
Four main factors influencing the anti-inflammatory, cardioprotective potential of the ketogenic diet. The above figure was created with BioRender.com. Accessed on 23 July 2023 Agreement number: ZI25N6RYI0.

Taking into account the proven anti-inflammatory effects of the ketogenic diet, its beneficial effects on the prevention or treatment of cardiovascular diseases seem reasonable. There are many potential mechanisms through which the ketogenic diet demonstrates its anti-inflammatory potential. However, four main factors can be identified. Firstly, the ketogenic diet places the body into a state of nutritional ketosis (associated, in turn, with a number of different biochemical and physiological mechanisms). The processes occurring during the state of ketosis exert a systemic anti-inflammatory effect, which in turn has a direct bearing on cardiovascular diseases. The second most important factor is the elimination of pro-inflammatory simple sugars from the diet. This is directly reflected within CVD. Both of these factors are described in detail in Section 3.1 and Section 3.2. In fact, the restriction of the total amount of carbohydrates in the diet can show specific anti-inflammatory benefits in the context of cardiometabolic health, as demonstrated in Section 3.3. A high-fat, well-composed ketogenic diet is also rich in omega-3 fatty acids, and their anti-inflammatory and cardioprotective effects are well known, as described in Section 3.4. The main factors are also illustrated in Figure 1.

### 3.1. Anti-Inflammatory, Cardioprotective Potential of the State of Ketosis (Ketone Bodies)

The ketogenic diet initiates the increased production of ketone bodies (i.e., β-hydroxybutyrate (the main ketone body), acetone and acetoacetate) in the body. Thus, it places the body into a state of nutritional ketosis. In view of this, the body uses ketone bodies, not glucose, as the main energy source for vital processes [79]. Ketone bodies and the state of ketosis exhibit systemic anti-inflammatory effects. Thus, a multifaceted anti-inflammatory effect is recognized, including in neurological diseases [80], cancer [81], diabetes [82], inflammatory bowel disease [83], the relief of chronic pain [84], the alleviation of the severity of chronic inflammatory disorders associated with obesity [85] and, finally, cardiovascular diseases [86].

The main ketone body, β-hydroxybutyrate (BHB), exhibits a number of anti-inflammatory properties, including mimicking the fasting state. It has multifaceted effects, including influencing gene expression, reducing inflammation and improving mitochondrial function. This is extremely important given that an integral aspect of cardiovascular diseases is, among others, inflammation. The primary anti-inflammatory effect of BHB is its inhibitory effect on the NLRP3 inflammasome, which is a type of command center for pro-inflammatory cytokines. The fact that it is sensitive to imbalance factors means that its activation may be associated with an increase in inflammatory markers [19]. At the same time, it is known that the NLRP3 inflammasome plays an important role in the heart, as its activation contributes to the deterioration and loss of myocardial function and the pathological development of cardiovascular diseases [87,88,89]. However, a study by Poff et al. showed that the administration of ketone bodies alone was effective in reducing a number of pro-inflammatory cytokines, including IL-1β, IL-6, IFN-γ, MCP-1 and RANTES. This demonstrates that ketone bodies have important anti-inflammatory effects [90]. The publication by Youm et al. confirms that BHB has the potential to alleviate inflammatory diseases mediated by the NLRP3 inflammasome. It inhibits the activation of the NLRP3 inflammasome in response to urate crystals, ATP and lipotoxic fatty acids. This occurs without oxidation in the TCA cycle and independently of uncoupling protein-2 (UCP2), sirtuin-2 (SIRT2), G-protein-coupled receptor GPR109A or hydrocarboxylic acid receptor 2 (HCAR2). The authors of the above publication further demonstrated that β-hydroxybutyrate reduced interleukin (IL)-1β and IL-18 production via the NLRP3 inflammasome in human monocytes [91]. With this in mind, BHB can contribute to the maintenance of normal cardiac function by inhibiting the NLRP3 inflammasome. Hydroxycarboxylic acid receptor 2 (HCAR2) may also be an important therapeutic target for the treatment of inflammatory disease processes. This is because it mediates anti-inflammatory effects in various tissues. It appears that BHB, through the binding and activation of HCAR2 or through the direct modulation of certain intracellular signaling pathways, has the ability to inhibit inflammatory responses and immune cell function [92,93,94]. Furthermore, a study by Shimazu et al. showed that β-hydroxybutyrate significantly protected against oxidative stress, which was associated with increased FOXO3A and MT2 activity. Indeed, BHB is a specific inhibitor of class I histone deacetylase (HDAC). The authors showed that HDAC inhibition by BHB was correlated with global transcriptional changes, including genes that encode oxidative stress resistance factors (FOXO3A and MT2). BHB was observed to increase histone acetylation in the Foxo3a and Mt2 promoters, and both genes were activated by the selective depletion of HDAC1 and HDAC2 [95]. Another study also showed that BHB inhibited the growth of ER-stress-related marker proteins and the inflammasome. Additionally, it was observed that BHB increased the expression of manganese superoxide dismutase and catalase through the transcription factor O3α pathway of AMP-activated protein kinase. This was applicable both in vivo and in vitro [96]. Ketone bodies in nutritional ketosis concentrations (as opposed to concentrations in ketoacidosis) also exert direct beneficial effects on vascular endothelial modulation, showing anti-inflammatory effects in the endothelium, among others [97]. Yurista et al., in their publication, explicitly stated that there is ample evidence that ketone bodies can directly inhibit inflammation in a beneficial manner in the context of cardiovascular diseases [98].

### 3.2. Anti-Inflammatory, Cardioprotective Effects of Elimination of Simple Sugars

The ketogenic diet involves limiting the total supply of carbohydrates, most often to a maximum of 50 g per day. In view of this, simple sugars are marginalized and, in fact, can often even be considered to be completely eliminated. This is important to minimize frequent increases in serum glucose and insulin, which inhibit the achievement of the full desired state of ketosis [19,99].

It is well known that simple sugars are one of the key pro-inflammatory dietary factors [100,101,102], which applies mainly to all simple sugars (by the manufacturer, consumer or food preparer). Concerns about the negative effects of simple sugars on CVD date back to the 1960s [103]. At the same time, however, low-fat diets were promoted for the prevention of CVD. Increasing the proportion of carbohydrates (including simple sugars) in the diet consequently led to worsening parameters related to cardiovascular health. It was only decades later that this was proven not to be a useful approach in the fight against increasing heart disease [104,105,106,107,108,109]. Too much added sugar in the diet can be one of the greatest threats to cardiovascular health. It exacerbates chronic inflammation, increasing the risk of developing CVD [110]. The negative effect of simple sugars was also shown by the authors of a large meta-analysis in 2022. They found that added sugar (as a percentage of daily energy intake) at ≥15.0% was positively correlated with total CVD (HR = 1.08 [1.01; 1.15]) and ischemic heart disease (CHD) (HR = 1.20 [1.09; 1.32]) [111]. In a 2023 publication, the authors also showed that free sugar (added sugar) intake was positively associated with total CVD (HR; 95% CI per 5% of energy, 1.07; 1.03–1.10), ischemic heart disease (1.06; 1.02–1.10) and stroke (1.10; 1.04–1.17). 

It should be clearly noted that the source of simple sugars often plays an important role. Free sugars are the most pro-inflammatory (including fructose in the form of sugar sweetened beverages (SSB), not necessarily from fruit). Kelly et. al. showed that replacing 5% of the energy from free sugars with non-free sugars was associated with a lower risk of total CVD (0.95; 0.92–0.98; *p*-trend = 0.001) and total stroke (0.91; 0.86–0.97; *p*-trend = 0.005) [112]. Studies show that the simple sugars present in fruit are unlikely to have a pro-inflammatory effect. A diet rich in fruits and vegetables may even help to reduce inflammation as they are a rich source of antioxidants and other bioactive substances [113]. It is known that a diet with excess fructose is pro-inflammatory, increasing the risk of metabolic syndrome or gout. Elevated levels of fructose metabolites (including uric acid and lactate) are closely related to oxidative stress and local inflammatory reactions in tissues and organs [114,115]. Although this concerns mainly added fructose (e.g., in the form of glucose or fructose syrup) and not whole fruit, there is evidence of a similar effect of fruit juices, especially in the context of increasing the risk of gout [116,117]. The ketogenic diet, however, marginalizes all forms of simple sugars (as they can “kicked out” from the ketosis state particularly easily) to a much greater extent than other nutrition models (which, according to the guidelines, allow, e.g., 5% free sugars in the diet) [118].

Glycated hemoglobin (HbA1c), which reflects the average serum glucose concentration over the past 3 months, is also one of the most important CVD risk factors [119]. Importantly, high HbA1c levels are strongly associated with CVD risk in people both with and without diabetes [120,121]. HbA1c has been shown to be positively correlated with CVD, such as carotid and coronary atherosclerosis, ischemic heart disease, ischemic stroke and hypertension, among others. The author of the publication also points out that HbA1c causes dyslipidemia, hyperhomocysteinemia and hypertension. In addition, it increases C-reactive protein (CRP) levels, oxidative stress and blood viscosity. All of these may eventually lead to the development of CVD [122]. It appears that HbA1c is an independent risk factor for the development of CVD and death from these diseases, even in people without diabetes. This shows the remarkable relevance of this marker for the general population [121]. However, the ketogenic diet has been shown to have HbA1c-lowering properties, through which it can also benefit CVD prevention and therapy. The efficacy of KD in lowering HbA1c is supported by a number of meta-analyses and other publications. The authors of the 2022 publication showed an average reduction in HbA1c of 1.45% in patients on a ketogenic diet (compared to those on control diets) [123]. Another meta-analysis from 2022 also showed a remarkable benefit of the ketogenic diet in lowering HbA1c. Patients on the ketogenic diet, compared to those on standard recommended diets, had reduced HbA1c levels after three and six months (by an average of 6.7 mmol/L and 6.3 mmol/L, respectively). Importantly, an advantage of the ketogenic diet over standard diets was observed even in lowering triglycerides and increasing HDL cholesterol [56]. Another meta-analysis in 2022 showed the effect of the ketogenic diet in lowering HbA1c (by an average of 0.38% HbA1c) and triglycerides (by an average of 0.36 mmol/L) and increasing HDL cholesterol (by an average of 0.28 mmol/L) [124]. Choi et al., in a meta-analysis, also showed greater benefits of the ketogenic diet compared to low-fat diets, among others, precisely in terms of a lower HbA1c concentration (SMD −0.62), increase in HDL concentration (SMD 0.31) and decrease in TG concentration (SMD −0.45) [125]. The beneficial effect of the ketogenic diet on glycated hemoglobin, triglycerides and HDL cholesterol values was also described in another meta-analysis from 2022 [126]. With the above in mind, it can be concluded that this is another factor by which the ketogenic diet has an anti-inflammatory effect, as it eliminates pro-inflammatory simple sugars.

### 3.3. Anti-Inflammatory, Cardioprotective Effects of Total Carbohydrate Restriction

The beneficial above-mentioned effects of ketogenic diets on the values of inflammatory markers and CVD risk factors may also be due to the reduction of the total carbohydrate pool, not simply the elimination of simple sugars. This may be supported by an extensive meta-analysis from 2022, which did not look strictly at the ketogenic diet but precisely at the effect of reducing the percentage of carbohydrates in the diet. It examined the effect of reducing the percentage of energy from carbohydrates from 55–65% to 10% on cardiometabolic risk factors in people with type 2 diabetes mellitus (T2DM). It was shown that each 10% reduction in carbohydrate energy percentage reduced the HbA1c concentration (by an average of 0.20 HbA1c%), fasting blood glucose (by an average of 0.34 mmol/L), triglyceride concentration (by an average of 0.12 mmol), body weight (by an average of 1.44 kg) and even systolic blood pressure (by an average of 1.79 mmHg). These values decreased linearly with a decrease in carbohydrate intake from 55–65% to 10%. These results reflected a 6-month period. When the indices were rechecked 12 months after baseline, HbA1c values continued their linear downward trend (by an average of 0.11 HbA1c%), as did triglyceride levels (a reduction of 0.12 mmol on average) [127]. At the same time, a U-shaped effect was observed at 6 months of follow-up for total and LDL cholesterol, where the greatest benefits occurred when reducing the amount of carbohydrates to 40% of energy, and for body weight at 12 months of observation (greatest benefits at 35% of carbohydrates within total energy). The authors indicated that the effect of limiting carbohydrates over a 12-month perspective was limited to HbA1c, body weight, LDL cholesterol and TG, with the effect size well below minimal clinically important difference (MCID) thresholds. Evidence for the cardiometabolic benefits of carbohydrate restriction alone can be found in a study by Waldman et al. The authors investigated the effects of a 4-week non-carbohydrate diet, with carbohydrate restriction to 25% of energy content, on markers of inflammation and oxidative stress among firefighters. The authors showed that this diet resulted in dramatic improvements in metabolic markers in the form of reductions in advanced oxidation protein products (AOPP) (51.3 ± 27.3 vs. 32.9 ± 7.9 ng-ml^−1^), malondialdehyde (MDA) (1.6 ± 0.6 vs. 1.1 ± 0.5 µmol-L^−1^) and triglycerides (84.4 ± 34.4 vs. 64.2 ± 14.4 mg-dL^−1^) [128]. Importantly, Karimi et al. showed that among a group of women studied, total dietary carbohydrate intake was associated with an increased risk of inflammation, whereas total fat intake was not associated with higher inflammation [129]. A reduction in inflammatory markers as a result of a low-carbohydrate diet was also cited by Tavakoli et al. [130]. Forsythe et al., in a randomized controlled trial, compared the effects of a low-carbohydrate diet and a low-fat diet for 12 weeks on markers of inflammation and circulating fatty acid composition. The authors concluded that the low-carbohydrate diet caused profound changes in fatty acid composition and reduced inflammation compared to the low-fat diet [131]. In another study, the authors also examined the effect of a carbohydrate-restricted diet on markers of cardiovascular disease for 12 weeks. They found that, after 12 weeks, CRP (−8.1%) and TNF-α (−9.3%), among others, decreased independently of weight loss. Body weight was reduced (−7.5 ± 2.5 kg); in addition, a decrease in plasma Lp(a) was observed (−11.3%). The authors concluded that carbohydrate restriction led to spontaneous calorie reduction and subsequent improvement in CVD markers in overweight or obese men [132]. The beneficial effect of carbohydrate restriction on direct cardiovascular risk factors was also supported by a large meta-analysis from 2020 [133]. 

It should be taken into account that the source of total carbohydrates itself is extremely important. Some studies do not take into account the source, while this certainly influences the subsequent results of the study. It is clear that a diet based on refined carbohydrates will be significantly worse compared to a diet based on unprocessed, whole grain carbohydrate sources. Given these relationships, in addition to achieving a state of ketosis and eliminating simple sugars, the ketogenic diet may also show beneficial anti-inflammatory cardioprotective potential as a result of limiting the total carbohydrate supply, especially if processed carbohydrates are subjected to this restriction.

### 3.4. Anti-Inflammatory, Cardioprotective Effects of Omega-3 Fatty Acids

A properly composed ketogenic diet is abundant in anti-inflammatory fatty acids from the omega-3 group. In this respect, it may show an advantage over other diets, especially low-fat diets. This is because it is much easier to provide the correct amounts of fatty acids, as one of the main foods is oily fish (which is the main source of omega-3). It is a high-fat diet, so there is much less concern about exceeding the percentage of energy from fats than with other diets.

Omega-3 fatty acids exhibit systemic anti-inflammatory effects, although, in the context of cardiovascular health, they are of particular importance. Indeed, it has been shown that omega-3 PUFAs, by competing with omega-6 PUFAs and displacing arachidonic acid in membrane phospholipids, exert anti-inflammatory properties by reducing the production of pro-inflammatory eicosanoids. Simonetto et al., in a publication, indicated that omega-3 PUFA supplementation may reduce the risk of various phenotypes of atherosclerosis and cardiovascular diseases [134]. A 2023 systematic review indicated and confirmed that omega-3 also improves the blood lipid profile [135]. The cardioprotective effect of omega-3 fatty acids is therefore firmly established in the literature and further confirmed by a number of recent publications, including meta-analyses [136,137,138,139,140].

Studies have shown that a ketogenic diet enriched in omega-3 fatty acids has an additional improved health-promoting effect. A randomized study by de Louis et al. showed that a very low-calorie ketogenic diet with additional docosahexaenoic acid (DHA) (and therefore omega-3) had a significantly better anti-inflammatory effect [141]. A 2022 study showed the effect of combining a ketogenic diet with additional amounts of omega-3 fatty acids. It showed improved metabolic profiles, improvements in hunger and satiety hormones, a large loss of body fat and, importantly, no effect on lean body mass. Reductions in CRP, total cholesterol, triglycerides, insulin and the HOMA-IR index, among others, were observed [142]. Another study indicating additional benefits of omega-3 fatty acid supplementation was published by Liu et al. in 2022. It showed that the combination of a low-carbohydrate and high-fat diet with additional omega-3 fatty acids improved lipid metabolism and aided weight control [143]. This is especially relevant as an N-3-enriched KD exerts better anti-inflammatory effects than KD alone [52]. 

Given that, inter alia, fatty fish (which is the main source of omega-3) is one of the main foods consumed in a ketogenic diet, it will thus be abundant in omega-3 fatty acids. In view of this, it represents another anti-inflammatory, cardioprotective factor that may result from a ketogenic diet. 

## 4. Ketone Bodies and Cardiac Energy Metabolism

The influence of the ketogenic diet on cardiac energy metabolism is increasingly being studied and described in scientific publications. This is due to the specific state of ketosis, in which there is increased induction of ketone body production. The main ketone body found in the blood, β-hydroxybutyrate, is seen to have the potential to affect myocardial metabolism and function. Of all the organs, the heart has the highest energy requirement. This is due to the need to work continuously, from fetal life until death. Thus, the cells of the heart muscle (cardiomyocytes) are characterized by the highest concentrations of mitochondria, i.e., “cellular power plants”, in the body. Indeed, mitochondria are the energy centers of the cells, as they are responsible for the production of energy in the form of adenosine triphosphate (ATP) [144,145]. It is known that in order to obtain ATP, the heart can use acetyl-coenzyme A (acetyl-CoA) from glucose (via glycolysis) or lipids (via β-oxidation). Under normal conditions, acetyl-CoA from fatty acids is the preferred substrate for ATP production in the heart. However, in patients with HFrEF, the contribution of ketone oxidation to myocardial ATP production increases from 6.4% (in control subjects) to 16.4% [146]. It appears that ketone bodies are therefore a very good, and, in many cases, perhaps better, energy substrate for the acquisition of ATP [97,147]. In the early stages of heart disease, the organ shifts its energy preference from fatty acids towards glucose, which is associated with a loss of metabolic flexibility [98,148]. However, it is still difficult to determine whether metabolic abnormalities occur due to the onset of heart disease or whether heart disease occurs as a result of metabolic abnormalities [149]. The fact is, however, that this eventually leads to heart failure, resulting in the further reprogramming of the heart’s metabolism towards the uptake and use of ketone bodies as an energy source. The fact that this may be an adaptive response offers a convincing argument for the relevance of ketone bodies to the metabolism of this organ. This is supported by a number of publications that show higher concentrations of ketone bodies and greater uptake of ketone bodies, among others, when heart failure occurs [150,151,152,153,154]. The heart prefers ketone bodies to glucose under conditions where both substrates are available. This was shown in a study by Gormsen et al., in which increasing the concentration of ketone bodies to 3.8 mM resulted in a 50% reduction in myocardial glucose uptake. This occurred despite maximal insulin stimulation and sufficient glucose [155]. It appears that the increased oxidation of ketone bodies is of particular use to the heart (and brain), as hyperketonemia does not affect glucose and fatty acid uptake in other organs [156,157]. A positive relationship has been observed between increased cardiac energy expenditure and levels of BHB and acetone, two ketone bodies [158]. Horton et al. demonstrated the cardiac relevance of ketone bodies using the example of the hearts of Cre-lox BDH1-KO mice, which lacked D-β-hydroxybutyrate dehydrogenase (BDH1), the mitochondrial enzyme responsible for the oxidation of ketone bodies (it catalyzes the first step in the oxidation of 3-hydroxybutyrate (3OHB)) [150]. It appeared that mice without BDH1 showed worsened heart failure in response to fasting or pressure overload/ischemia compared to mice with BDH1. At the same time, increased hydroxybutyrate administration improved pathological cardiac remodeling and dysfunction, as well as the bioenergetic thermodynamics of isolated mitochondria, during reduced fatty acid utilization. The authors indicate that the heart increases the utilization of ketone bodies in response to metabolic stress. It is directly suggested that the administration of ketone bodies may be an important component of heart failure therapy. Another study showed that the concentration of all ketone bodies was almost twice as high in dogs with heart failure compared to control dogs. In addition, the administration of a cardioprotective protein similar to folistatin 1 (FSTL1) reduced the uptake of ketone bodies [159]. While most research focuses on the effect of exogenous ketones on the energy metabolism of the heart, there are also publications examining the direct effect of the ketogenic diet itself. Interesting results were reported in the 2022 study by Guo et al. This is because it showed that a ketogenic diet taken every other day was protective against heart failure by showing a strong cardioprotective effect. However, no protective properties against heart failure were demonstrated during the continuous use of a ketogenic diet for 8 weeks. The authors suggested that while continuous use of the ketogenic diet impaired hepatic ketogenesis capacity, when used every other day, it preserved the hepatic ketogenesis capacity [160]. A 3.2-year-long population-based study from 2017 showed an association of increased BHB levels with an increased risk of cardiovascular events in 405 hemodialyzed elderly patients. The authors indicated that elevated BHB levels were independently associated with cardiovascular events and death from any cause among the studied group of patients [161]. While no differences in outcomes were observed between the two sexes, differences were shown by another, larger population-based study. Flores-Guerrero et al. showed that high plasma BHB levels were associated with an increased risk of heart failure with reduced ejection fraction (HFrEF), particularly among women [162]. The results obtained in both studies may give the primary impression of causality. Meanwhile, as has been shown in previous publications, in heart failure (and other cardiac lesions), the concentration of ketone bodies increases and this should be considered more in the category of “rescue fuel”. The results/observations, therefore, provide confirmation that ketone bodies are extremely important for the function of the diseased heart. This is supported by a 2023 publication [163]. The study by Kashiwagi et al. also showed that B-type natriuretic peptide (BNP) can induce elevated concentrations of ketone bodies for use as an important alternative fuel in the failing heart. The authors indicated that higher concentrations of ketone bodies were more strongly stimulated by BNP than by hemodynamic deterioration. This was due to the observation that there was a positive correlation between ketone bodies and BNP concentrations, but not between ketone bodies and left ventricular end-diastolic pressure (LVEDP), the left ventricular end-systolic volume index (LVESVI) and the left ventricular end-diastolic volume index (LVEDVI) [164]. In addition to the presence of higher β-hydroxybutyrate concentrations in patients with heart failure, this was also manifested by increased amounts of acetone (one of the ketone bodies) in the exhaled air of such individuals. One study indicated that patients with HFrEF had elevated levels of acetone and, importantly, these were inversely related to heart function. The authors indicated that high exhaled acetone levels may be associated with poor prognosis in patients with HFrEF [165]. In a post hoc analysis of 79 patients with acute HF participating in the EMPA-RESPONSE-AHF trial, it was shown that levels of ketone bodies, particularly acetone, were significantly elevated during an episode of acute decompensated heart failure compared with after stabilization [166]. In a randomized controlled trial, Nielsen et al. showed that the administration of 3-hydroxybutyrate (3-OHB) in patients with heart failure increased the cardiac minute volume by 2.0 ± 0.2 L/min. There was an increase in stroke volume by 20 ± 2 mL and heart rate by 7 ± 2 beats per minute (BPM). In addition, the left ventricular ejection fraction increased by 8 ± 1%. The authors concluded that an increase in serum BHB in the physiological range had beneficial hemodynamic effects in patients with HFrEF without impaired myocardial external efficiency (MEE) [167]. Nasser et al. indicated, on the basis of the cited studies [151,152,168,169,170], that ketone bodies (derived from a ketogenic diet) may improve myocardial function and contribute to the more effective treatment of patients with cardiovascular dysfunction [97]. There are also a number of other pieces of evidence for the remarkable relevance of ketone bodies to cardiac function, as described by, among others, Abdul et al. [147]. The conclusions of another publication suggest that the ketogenic diet is an intriguing non-pharmacological option for the treatment and prevention of cardiovascular diseases, particularly heart failure [171]. A 2023 publication indicates that increasing evidence supports an adaptive role for ketone metabolism in heart failure to promote normal heart organ function and mitigate disease progression [172]. Medium-chain fatty acids (MCTs) also play an important role in ketogenic diets, the addition of which can be considered the highest standard for ketogenic diets [173]. This is because they are the most ketogenic fatty acids among all other sources. They are easily digested and result in the more rapid production of ketones compared to long-chain fatty acids. MCTs represent “fast energy” because, unlike long-chain fatty acids, they do not require pancreatic enzymes for digestion and bypass the standard pathway through the gastrointestinal tract, traveling via the portal vein to the liver and providing a rapid source of energy or converting into ketones [19]. Given the need to supply energy to a constantly working heart and the speed at which MCTs do so, as well as their high ketogenicity, they may be a beneficial part of the diet, especially when combined with a ketogenic diet (with which they are often combined). This is of even greater importance as MCTs have been shown to increase mitochondrial biogenesis and metabolism (which is present largely in cardiomyocytes), thereby improving performance during exercise [174]. Additionally, it was observed that among patients with coronary heart disease, the inclusion of coconut oil (which is a source of MCTs) increased the HDL cholesterol fraction and reduced the waist circumference [175]. With this in mind, the enrichment of the ketogenic diet with MCT fats is justified due to the multifaceted beneficial effects of MCTs. When analyzing all the evidence, it is worth noting that most publications describe the effect of exogenous ketones, or their increase in the case of cardiac cell metabolism disorders, and not the direct effect of the ketogenic diet. However, given the large amount of indirect evidence indicating the potential benefits of this model of nutrition, it is worth conducting specific clinical trials examining the direct impact of a strictly ketogenic diet on the parameters of energy metabolism in the heart.

## 5. The Ketogenic Diet and the Vascular Endothelium

**Figure 2:**
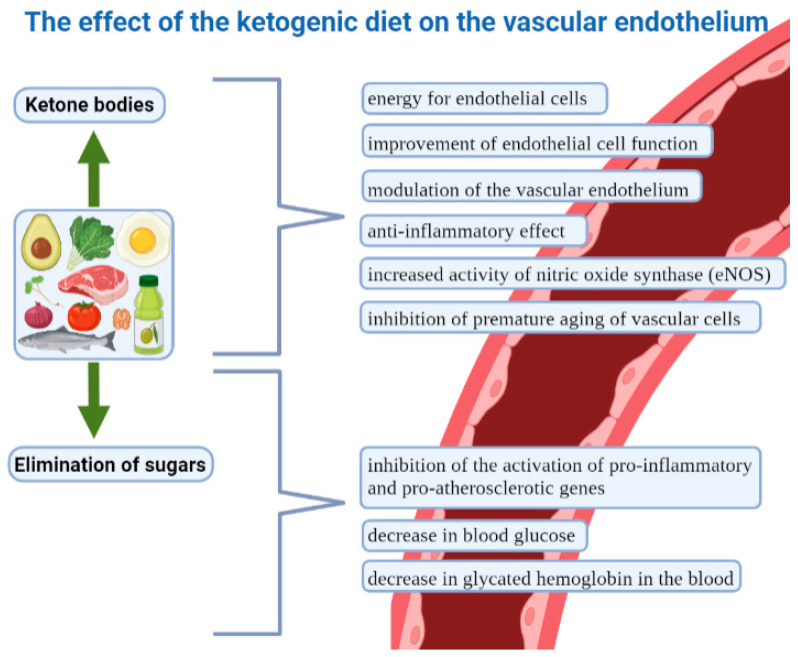
Influence of the ketogenic diet on vascular endothelial cells. The above figure was created with BioRender.com. Accessed on 23 July 2023. Agreement number: ZI25N6S5GU.

Endothelial cells are extremely important in maintaining the function of the cardiovascular system and, by extension, the whole body. They regulate vascular tone by, among other actions, producing nitric oxide, endothelin and prostaglandins. They produce and respond to various cytokines and adhesion molecules. In addition, they are key immunoreactive cells, and their dysfunction results in a number of pathological changes. In addition, they play an extremely important role in many other processes [176,177]. It appears that the ketogenic diet, via ketone bodies, can also affect endothelial cells. These cells are intimately involved in the transport of ketone bodies. Importantly, they are able to take them up and use them to generate biomass and ATP, as they express succinyl-CoA:3-oxoacid-CoA transferase (SCOT) (an enzyme that oxidizes ketone bodies). This was demonstrated in a 2022 publication showing that cardiac endothelial cells are capable of oxidizing ketone bodies, which increases proliferation, cell migration and vascular sprouting. Additionally, in a mouse model of cardiac hypertrophy, the ketogenic diet prevented vasodilation. In view of this, the ketogenic diet may play a beneficial role in heart disease [178]. The publication by Nasser et al. adds further evidence of the protective effect of ketone bodies. Ketone bodies (mainly BHB) in low concentrations (achievable with a ketogenic diet) have been shown to potentially improve endothelial and vascular function in metabolic disease. At the same time, excess ketone bodies resulting from diabetic ketoacidosis have been shown to affect diabetic vasculopathy and the vascular complications of diabetes [97]. However, diabetic ketoacidosis is known to occur with simultaneous excess concentrations of ketone bodies and glucose, with such high levels of ketone bodies not usually achievable through a ketogenic diet [19]. Unsurprisingly, diabetic patients experiencing ketoacidosis are at risk of not only vascular complications but even death from them [179,180,181]. However, ketone bodies in nutritional ketosis concentrations (induced by a ketogenic diet) exert direct beneficial effects on vascular endothelial modulation, showing anti-inflammatory effects in the endothelium, among others [97]. Gormsen et al. further demonstrated that hyperketonemia induced by Na-3-hydroxybutyrate infusion increased myocardial blood flow by up to 75% and heart rate by approximately 25%. The authors concluded that ketone bodies reduce myocardial glucose uptake and increase myocardial blood flow. They thus suggested that ketone bodies are important vasodilators and an important source of fuel for the heart. This affects the overall therapeutic potential of ketone bodies in CVD [155]. Another randomized controlled trial showed that the infusion of 3-hydroxybutyrate increased the cardiac minute volume by 2 L/min (40%), with an absolute improvement in the left ventricular ejection fraction (8%). Importantly, an effect on vasodilation was noted. This was accompanied by stable systemic and pulmonary pressure [167]. In an animal study, increased endothelial nitric oxide synthase (eNOS) protein expression was observed under the influence of the ketogenic diet. The authors concluded that KD may improve, among other factors, cerebral vascular function while improving the metabolic profile, increasing favorable gut microflora and reducing the risk of Alzheimer’s disease [182]. A study by McCarthy et al. further demonstrated that BHB stimulates the production of endothelium-derived factors. Indeed, the infusion of 1,3-butanediol (a precursor of BHB) increased nitric oxide synthase activity. The authors suggested that low concentrations of BHB may offer a new therapy for hypertension-associated vascular ageing by increasing nitric oxide synthesis [183]. In the context of vascular endothelial ageing, there is also a body of evidence demonstrating the beneficial, protective effects of ketone bodies on this process. Among other aspects, BHB has been shown to reduce the secretory phenotype associated with ageing and vascular cell senescence in mammals [184]. The results of a study by Han et al. indicated that BHB promotes vascular cell dormancy and that this significantly inhibits stress-induced premature ageing and replicative ageing through p53-independent mechanisms. Furthermore, among other outcomes, it upregulated Oct4 and Lamin B1 in both vascular smooth muscle and endothelial cells in mice. The authors concluded that BHB exerted anti-ageing effects in vascular cells through the upregulation of the Lamin B1 pathway induced by hnRNP A1, which was mediated by Oct4 [185]. The results of Meroni et al.’s work suggest that the ketogenic diet, mediated by the induction of moderate oxidative stress, activates the transcription factor Nrf2. This factor in turn induces the transcription of target genes involved in the cellular antioxidant defense system [186]. Glucose and HbA1c levels also decrease under a ketogenic diet [82]. At the same time, it is known that the chronic exposure of vascular endothelial cells to high glucose concentrations leads to the increased expression of several pro-inflammatory and atherosclerotic genes [187]. In view of this, the glucose-lowering mechanism is another factor that favors vascular endothelial function. On the other hand, there are also reports that indicate a deterioration in endothelial function. A study by Coppola et al. showed increased arterial stiffness in children with epilepsy treated with KD [188]. Another study indicated that cardiovascular risk may be increased, but only during the first few days of a very low-carbohydrate diet [189]. It is worth noting, however, that most research is still focused on the impact of exogenous ketones, not the ketogenic diet. However, considering the totality of the evidence, the vast majority attests to the beneficial effects of the ketogenic diet, and the ketone bodies induced through it, on vascular endothelial function. The effects of the ketogenic diet on the vascular endothelium are illustrated in Figure 2.

## 6. The Ketogenic Diet and Blood Pressure

Taking into account the nature of the ketogenic diet, it seems reasonable to consider how this dietary intervention affects blood pressure. Indeed, there are several mechanisms by which it is able to affect this parameter. On the one hand, it is known that the ketogenic diet reduces insulin concentrations because it provides a low amount of carbohydrates [82]. Insulin, on the other hand, is responsible for the retention of sodium in the body by stimulating its reabsorption, and this is also related to the retention of water in the body. With a decrease in insulin concentrations, there is then the increased removal of water and sodium from the body [190,191]. This in turn leads to the removal of more of the other electrolytes, e.g., potassium, with the urine [192,193]. Magnesium or calcium may also decrease. However, it has been shown that electrolyte loss only occurs during the first period of the ketogenic diet (first week) and is due to natural adaptations to the state of ketosis [19,194,195]. It is known that adequate electrolyte levels are an essential factor in maintaining normal blood pressure [196]. On the other hand, it is also known that the vascular endothelium has an extremely important influence on blood pressure [197,198], which can be affected by the ketogenic diet. In addition, it may beneficially affect the hypothalamic–pituitary–adrenal (HPA) axis and the sympathetic nervous system (SNS) [199,200]. Another possible mechanism for the effect on blood pressure may include the effect of the ketogenic diet on the renin–angiotensin–aldosterone (RAA) system, as discussed in two publications from 2023 [201,202]. Weight loss is a significant, simple mechanism involved in the influence of the ketogenic diet on blood pressure. A caloric deficit used simultaneously with the ketogenic diet, and weight loss itself (and an improvement in body composition), can indirectly improve blood pressure parameters [203,204,205]. A number of potential mechanisms for the effects of the ketogenic diet on blood pressure make it worthwhile to consider publications examining actual blood pressure changes that have occurred in people using this dietary intervention. The authors of a 2021 publication indicate that the ketogenic diet is able to provide a reduction in blood pressure but does not produce significant changes compared to non-ketogenic diets. They also indicate that this is largely due to weight and fat loss and improvements in CVD risk parameters [203,204,205]. A 2023 publication investigating the effect of a very low-calorie ketogenic diet (VLCKD) in women with obesity and hypertension found that both systolic and diastolic blood pressure improved significantly (−12.89% and −10.77%, respectively; *p* < 0.001). Before the dietary intervention, the mean systolic blood pressure was 140.88 ± 8.99 mmHg and the diastolic blood pressure was 88.90 ± 6.71 mmHg. After 45 days on the ketogenic diet, the systolic blood pressure decreased to an average of 122.56 ± 10.08 mmHg and the diastolic blood pressure to an average of 78.94 ± 6.68 mmHg. The authors, therefore, concluded that a very low-calorie ketogenic diet safely reduced blood pressure in women with obesity and hypertension [206]. Importantly, this was mainly due to a caloric deficit, weight loss and waist circumference, rather than the dietary pattern itself. There is a high probability that a similar effect could be obtained by using the same deficit in a well-balanced standard diet. Another study from 2023 also indicated that VLCKD was effective in reducing blood pressure in patients with non-alcoholic fatty liver disease (NAFLD) over an 8-week period. There was a reduction in the mean systolic blood pressure from 133.51 ± 12.86 mmHg to 123.27 ± 10.51 mmHg and the diastolic blood pressure from 81.73 ± 8.09 mmHg to 75.27 ± 7.84 mmHg [207]. In a 2022 randomized controlled trial, the ketogenic diet together with continuous positive airway pressure (CPAP) reduced systolic and diastolic blood pressure values to a greater extent (from 142.8 ± 13. 3 mmHg to 133 ± 11.9 mmHg and from 85.4 ± 8.38 mmHg to 78.7 ± 6.43 mmHg) compared to CPAP alone (from 134.2 ± 10.4 mmHg to 130 ± 9.7 mmHg and from 87 ± 11.6 mmHg to 82 ± 9.5 mmHg) in patients with severe obstructive sleep apnea syndrome [45]. Another randomized controlled trial compared the effects of different ketogenic diets, depending on the type of protein, on the parameters of patients with obesity and insulin resistance. It found that there was a significant reduction in blood pressure values in each ketogenic diet group. Systolic blood pressure changed on average in WPG from 132 ± 10 mmHg to 124 ± 13 mmHg, in VPG from 131 ± 8 mmHg to 121 ± 10 mmHg and in APG from 129 ± 9 mmHg to 121 ± 16 mmHg. Diastolic pressure decreased on average in WPG from 78 ± 11 mmHg to 70 ± 9 mmHg, in VPG from 78 ± 10 mmHg to 72 ± 10 mmHg and in APG from 78 ± 10 mmHg to 71 ± 9 mmHg [49]. In a randomized trial, Saslow et al. investigated the effect of a moderate- to very low-carbohydrate diet in overweight adults with type 2 diabetes or pre-diabetes. However, they found that there were no clear differences between the two groups [51], as described in detail in Table 2. Another RTC showed that the effects of a ketogenic diet and a hypocaloric diet on blood pressure were not significantly different. The mean systolic blood pressure decreased in those on the ketogenic diet from 110 ± 13 mmHg to 108 ± 13 mmHg, while the diastolic blood pressure increased from an average of 66 ± 10 mmHg to 68 ± 8 mmHg. A non-significant reduction in systolic blood pressure from 107 ± 9 mmHg to 106 ± 11 mmHg and diastolic blood pressure from an average of 65 ± 10 mmHg to 62 ± 11 mmHg was observed in those on the hypocaloric diet [53]. Yancy et al., in a subsequent RCT, compared the effects of a ketogenic diet with those of a low-fat diet combined with orlistat therapy (the drug used for obese patients at the time) on various parameters, i.e., body weight, blood pressure, fasting serum lipid levels and glycemic parameters. It was shown that those on the ketogenic diet had better results, including blood pressure. After 48 weeks, the mean systolic blood pressure decreased by −5.94 mmHg (−1.5 mmHg in LFD + O) and diastolic blood pressure decreased by −4.53 mmHg (−0.43 mmHg in LFD + O) [208]. Focusing strictly on comparative studies of low-carbohydrate vs. low-fat diets, it is worth citing the randomized controlled trial conducted by Foster et al. The study showed that a low-carbohydrate diet was more effective in lowering blood pressure compared to a low-fat diet. It was noted that at each stage (3, 6, 12 and 24 months), diastolic blood pressure decreased more (by 2 to 3 mmHg) in the low-carbohydrate diet group. There were no large differences between the two groups in the reduction in systolic blood pressure, although, after 3, 6, 12 and 24 months, the decrease was still greater in the low-carbohydrate group [209]. Another RCT showed that a ketogenic diet (up to 30 g of carbohydrate per day) without calorie restriction had the same non-significant effect on blood pressure reduction as a diet with a calorie deficit and a fat supply of up to 30% of energy [210]. This study also indicated that the caloric deficit itself was not the most important factor affecting blood pressure parameters. In a pilot study, Tzenios et al. showed that, after 140 days, a ketogenic diet reduced systolic blood pressure values by 5.3% from baseline. The authors also reported a significant increase in diastolic blood pressure at day 28; however, there was no significant change at days 56, 70, 84, 112 and 140 [211]. We can also note the results of a meta-analysis by Castellan et al., in which the authors indicated that VLCKD was associated with a mean reduction in systolic blood pressure of 9 mmHg and diastolic blood pressure of 7 mmHg, in addition to significant effects in terms of reducing body mass index (BMI) (−5.3 kg/m^2^), waist circumference (−12.6 cm), HbA1c (−0.7%), total cholesterol (−28 mg/dL), triglycerides (−30 mg/dL) and liver enzymes [60]. In another meta-analysis, the authors compared the effect of a ketogenic diet to a low-fat diet, among others, in terms of changes in blood pressure. They found that the ketogenic diet was more effective in lowering diastolic blood pressure (WMD—1–43 (95% CI—2–49, 0–37) mmHg), while differences were shown to a lesser extent in systolic blood pressure (WMD in favor of VLCKD—1–47 (95% CI—3–44, 0–50) mmHg) [212]. All the studies described are presented in Table 2.

Taking into account the results of the currently available studies, it can undoubtedly be concluded that the ketogenic diet may have a beneficial effect on blood pressure values. In addition to exerting a similar effective hypotensive effect to other weight reduction interventions, the ketogenic diet seems to show some advantage in this respect, which is not only due to weight loss. Further well-designed comparative studies are clearly needed to be able to achieve a single, definitive consensus in this regard.

## 7. The Ketogenic Diet and Weight Loss as a Factor in CVD Prevention and Therapy

The ketogenic calorie deficit diet is often used to reduce excess body weight. Taking into account its nature, for many people, it is a more effective weight loss strategy compared to standard diets. Taking into consideration that obesity is one of the primary risk factors for CVD, weight loss in itself will significantly reduce the risk of these diseases. Obesity, therefore, simultaneously is the effect of other risk factors, i.e., poor diet and physical inactivity, while at the same time being another risk factor on its own [1,6,59,213,214]. In addition, it affects other risk factors, e.g., dyslipidemia, hypertension, type 2 diabetes and sleep disorders, among others. Furthermore, an increased waist circumference can be considered as an independent risk factor for CVD, independent of BMI. Excessive visceral obesity is also an independent indicator and has been linked to poor cardiovascular outcomes. Losing excessive body weight thereby improves cardiovascular function, resulting in a reduction in CVD risk [215].

In addition to these mechanisms, the ketogenic diet improves CVD risk factors and, in addition, often reduces body weight (often the main reason for its use). Thus, it decreases the risk factor of obesity/overweight and, at the same time, nullifies other risk factors that arise from excess body weight. A meta-analysis by Bueno et al. showed that the ketogenic diet may be a more effective option for long-term weight loss (and improvements in some CVD risk factors), compared to low-fat diets [212]. The superiority of ketogenic diets over low-fat diets was also shown in a meta-analysis by Choi et al. It was shown that, compared to low-fat diets, the ketogenic diet was more effective in improving metabolic parameters related to, among other factors, body weight, lipid profile and glycemic control in patients with excess body weight (overweight or obesity), particularly in diabetic patients [125]. It appears that the ketogenic diet (a very low-calorie diet for weight loss) is not only effective in reducing body weight but is also safe to follow. This is confirmed by the results of another meta-analysis [60]. Taking this into account, weight loss is another factor by which the ketogenic diet may show preventive and therapeutic potential in cardiovascular diseases.

## 8. The Effect of the Ketogenic Diet among Patients with CVD and Healthy People

Some studies often present greater benefits of the ketogenic diet among CVD patients than among healthy and often physically active people. These differences may be due to certain mechanisms. Firstly, people suffering from CVD frequently also have excessive body weight (which is much less common in healthy people), so a greater weight loss effect is observed among people affected by CVD. Additionally, it is known that weight loss alone provides a marked improvement in parameters related to CVD risk [215]. This is shown e.g., in publications by Li et. al, Schiavo et. al. and Michalczyk et. al., in which people using the ketogenic diet lost significantly more body weight compared to the control group, thus improving the values of parameters such as total cholesterol, LDL, HDL and triglycerides [43]. This aspect is described in more detail in Section 7. 

The other mechanism is the fact that people suffering from CVD have, by definition, an abnormal lipid profile, which is not present to such an extent in healthy and physically active people. An example is a study in which, in healthy, young and trained women, the ketogenic diet did not improve their lipid profiles, and the authors even concluded that it deteriorated [47], which is in opposition to a number of studies described (in Section 2.2) in which the ketogenic diet reduced CVD risk parameters among patients with and without overweight. Another argument for the greater benefits of the ketogenic diet among people suffering from or at risk of CVD is the composition of the current diet.

The composition of the diet of these people is often inappropriate, particularly regarding the content of many processed products. A Western-style diet alone worsens CVD risk parameters, thereby increasing the risk of developing the disease. Therefore, the act of switching to an unprocessed diet (which, as a rule, should be a ketogenic diet) should reduce this risk. An example was given in a publication that compared the adoption of an unprocessed ketogenic diet to the continuation of a standard Western model of nutrition (which certainly had a significant impact on the body weight of the surveyed women and dysregulation of the lipid profile, insulin or glucose levels). Women who switched to a ketogenic diet showed significantly improvements in their overall health and CVD risk parameters [50]. Another important mechanism is the fact that healthy people may not experience such a significant impact of ketones (induced, among others, by a ketogenic diet) on the function of the heart, because, as has been repeatedly shown, heart cells increase the uptake of ketone bodies only in the case of impaired metabolism, which occurs in people with CVD [146,147,150,151,152,153,163]. Another mechanism may be the effect on the vascular endothelium itself. In healthy people who do not have problems with endothelial inflammation, it is also difficult to determine the potential benefits of, among others, ketone bodies. 

Furthermore, patients with CVD also struggle with chronic endothelial inflammation, which, research shows, can be reduced by raising ketone bodies to the levels achievable with a ketogenic diet [97,155]. In addition, reductions in the factors that influence endothelial inflammation, i.e., glucose, glycated hemoglobin and insulin concentrations (described in Section 3.2), also show significant differences related to the diet’s impact on people affected by CVD and on healthy people.

## 9. Conclusions

Summarizing the extensive scientific evidence, the ketogenic diet is a promising nutritional model in the context of cardiovascular disease prevention and therapy. Through its pleiotropic properties, it is able to influence the cardiovascular system on multiple levels. Scientific evidence mostly confirms its beneficial (even more beneficial compared to other diets) effects on the lipid profile and other CVD risk factors. However, there is a lack of strong evidence of the CVD risk from dyslipidemia due to the ketogenic diet. A potential advantage of the ketogenic diet is the strong anti-inflammatory effect that interacts with the cardioprotective properties. In addition, the effect on cardiomyocyte metabolism and the increased uptake of ketone bodies in cardiac disorders means that ketone bodies can be described as “rescue fuel” for the heart. The multifaceted effects of the ketogenic diet may also be confirmed by the effect of ketone bodies on the vascular endothelium, modulating vascular endothelial cells, improving their function or delaying their ageing. This also confirms the beneficial effect of the ketogenic diet on blood pressure values and other indirect CVD risk factors, i.e., reduction in excess body weight. A number of these factors contribute to the overall cardioprotective potential of the ketogenic diet in the prevention and treatment of cardiovascular diseases. This is confirmed by an increasing number of recent scientific studies.

Taking into account that cardiovascular diseases are a major (and increasing) cause of death worldwide, it will be of the utmost importance to meticulously analyze and review the current management and knowledge in this area. The current scientific evidence on the impact of the ketogenic diet in CVD prevention and therapy is optimistic. Taking this into consideration, there is a legitimate need for further scientific research on the relationship between KD and CVD. This could contribute to improving health and reducing the risk of death among many millions of people worldwide.

## Figures and Tables

**Table 1:** The effect of the ketogenic diet on the blood lipid profile.

Research Type, Year	Purpose of the Study	Type of Diet	Changes in the Lipid Profile	References
RCT, 2022	Observation of periodic ketogenic diet for effect on overweight or obese patients newly diagnosed as T2DM.	Ketogenic diet (KD) vs. standard diabetes diet (SDD)	KD vs. SDD: -decrease in cholesterol from 4.54 ± 0.69 mmol/L to 4.02 ± 0.43 mmol/L (SDD from 4.56 ± 0.67 mmol/L to 4.23 ± 0.47 mmol/L) -decrease in triglycerides from 1.76 ± 0.59 mmol/L to 1.44 ± 0.26 mmol/L (SDD from 1.81 ± 0.78 mmol/L to 1.66 ± 0.46 mmol/L) -decrease in LDL from 2.75 ± 0.65 mmol/L to 2.34 ± 0.45 mmol/L (SDD from 2.77 ± 0.69 mmol/L to 2.59 ± 0.58 mmol/L) -increase in HDL from 1.08 ± 0.11 mmol/L to 1.21 ± 0.23 mmol/L (SDD from 1.09 ± 0.19 mmol/L to 1.12 ± 0.20 mmol/L)	[43]
RCT, 2022	Comparison of 2 low-carbohydrate diets with 3 key similarities and 3 key differences for their effects on glucose control and cardiometabolic risk factors in individuals with prediabetes and T2DM.	Well-formulated ketogenic diet (WFKD) vs. the Mediterranean-plus diet (Med-Plus)	WFKD vs. Med-Plus: -reduction in triglycerides from 118.8 mg/dL to 99.5 mg/dL (in Med-Plus from 131.1 mg/dL to 121.7 mg/dL) -increase in HDL concentration from 49.1 mg/dL to 54.1 mg/dL (in Med-Plus from 48 mg/dL to 47.9 mg/dL) -increase in LDL concentration from 97.8 mg/dL to 111.3 mg/dL (in Med-Plus from 111.5 mg/dL to 95.3 mg/dL)	[44]
RCT, 2022	Assessment of the clinical advantage of combining two preoperative strategies (continuous positive airway pressure (CPAP) and low-calorie ketogenic diet (LCKD)) compared to CPAP alone, to improve apnea–hypopnea index (AHI) score, hypertension (HTN), dyslipidemia (DLP), insulin resistance (IR) and C-reactive protein (CRP) levels in patients with severe obesity and obstructive sleep apnea syndrome (OSAS) scheduled for bariatric surgery (BS).	Low-calorie ketogenic diet (LCKD) + continuous positive airway pressure (CPAP) vs. only continuous positive airway pressure (CPAP)	LCKD + CPAP vs. CPAP: -reduction in total cholesterol from 200.1 ± 30.1 mg/dL to 180.4 ± 35.2 mg/dL (CPAP from 196.1 ± 32.9 mg/dL to 180.8 ± 33.0 mg/dL) -decrease in LDL from 127.4 ± 26.8 mg/dL to 107.1 ± 37.1 mg/dL (CPAP from 128 ± 30.2 mg/dL to 112.9 ± 34.9 mg/dL) -decrease in triglycerides from 191 ± 41.7 mg/dL to 130 ± 79 mg/dL (CPAP from 151.6 ± 62.5 mg/dL to 129.7 ± 62.2 mg/dL) -insignificant increase in HDL from 48.3 ± 9.41 mg/dL to 48.8 ± 10.4 mg/dL (CPAP from 46.4 ± 10.3 mg/dL to 47.3 ± 9.8 mg/dL)	[45]
RCT, 2021	Investigation and comparison of the effects of two iso-energetic hypo-caloric ketogenic hyper-ketonemic and non-ketogenic low-carbohydrate high-fat high-cholesterol diets on body-composition, muscle strength and hormonal profile in experienced resistance-trained middle-aged men.	Ketogenic diets (KD) vs. non-ketogenic diets (NKD) (in several variants)	No significant differences in lipid profile. -In KD—change in TC from 4.44 ± 0.37 mmol/L to 4.43 ± 0.30 mmol/L (NKD from 4.49 ± 0.31 mmol/L to 4.52 ± 0.30 mol/L) -In KD—change in TG from 0.99 ± 0.25 mmol/L to 0.95 ± 0.26 mmol/L (NKD from 0.90 ± 0.14 mmol/L to 0.85 ± 0.13 mmol/L) -In KD—change in HDL from 1.28 ± 0.13 mmol/L to 1.36 ± 0.12 mmol/L (NKD from 1.28 ± 0.13 mmol/L to 1.36 ± 0.12 mmol/L) -In KD—change in LDL from 2.40 ± 0.21 mmol/L to 2.45 ± 0.25 mmol/L (NKD from 2.57 ± 0.41 mmol/L to 2.59 ± 0.39 mmol/L)	[46]
RCT, 2021	Investigation of the effect of a ketogenic LCHF diet on low-density lipoprotein (LDL) cholesterol (primary outcome), LDL cholesterol subfractions and conventional cardiovascular risk factors in the blood of healthy, young, and normal-weight women.	Ketogenic low-carbohydrate high-fat (LCHF) diet vs. National Food Agency recommended control diet (NFACD)	The LCHF diet: -increases in LDL cholesterol in every woman with a treatment effect of 1.82 mM (*p* < 0.001) (primary outcome at baseline = 2.1 ± 0.6 mM) -increases in apolipoprotein B-100 (ApoB) (treatment effect (95% Cl) = 0.50 [0.35, 0.65], primary outcome at baseline = 0.70 ± 0.15 g/L) -increases in LDL 1–2 (large, buoyant LDL) (treatment effect (95% Cl) = 31.56 [21.60, 41.51], primary outcome at baseline = 42.1 ± 14.6 mg/dL) -increases in LDL 3–7 (small, dense LDL) (treatment effect (95% Cl) = 4.51 [1.87, 7.16], primary outcome at baseline = 2.7 ± 2.5 mg/dL)	[47]
RCT, 2020	Comparison of the efficacy, safety and effect of 45-day isocaloric very-low-calorie ketogenic diets (VLCKDs) incorporating whey, vegetable or animal protein on the microbiota in patients with obesity and insulin resistance, to test the hypothesis that protein source may modulate the response to VLCKD interventions.	Isocaloric VLCKD regimens (≤800 kcal/day) containing whey (WPG), plant (VPG) or animal protein (APG)	Significant reductions in total cholesterol (TC), LDL and triglycerides (TG) in all VLCKD groups: -TC in WPG from 214.8 ± 31.5 mg/dL to 166.2 ± 43.6 mg/dL, in VPG from 220.9 ± 51.6 mg/dL to 170.7 ± 36.3 mg/dL, in APG from 226.9 ± 32.7 mg/dL to 191.2 ± 34.2 mg/dL -LDL in WPG from 132.8 ± 30.8 mg/dL to 100.8 ± 38.4 mg/dL, in VPG from 136.1 ± 41.3 mg/dL to 97.5 ± 32.3 mg/dL, in APG from 143.9 ± 25.8 mg/dL to 118.5 ± 23.1 mg/dL -TG in WPG from 131.0 ± 44.9 mg/dL to 94.6 ± 32.0 mg/dL, in VPG from 170.1 ± 126.9 mg/dL to 117.6 ± 42.7 mg/dL, in APG from 124.25 ± 58 mg/dL to 82.25 ± 33.32 mg/dL -insignificant changes in HDL: in WPG from 51.7 ± 12.3 mg/dL to 46.1 ± 7.5 mg/dL, in VPG from 51.2 ± 12.8 mg/dL to 49.0 ± 9.5 mg/dL, in APG from 57.9 ± 23.7 mg/dL to 56.2 ± 18.0 mg/dL	[49]
RCT, 2020	Comparison of the influence of a 12-week, well-planned, low-calorie ketogenic diet (LCKD) on hyperglycemic, hyperinsulinemic and lipid profiles in adult, overweight or obese females.	Low-calorie ketogenic diet (LCKD) vs. control group (CG) (typical diet)	Significant reduction in TG and increase in HDL in LCKD compared to CG: -TG in LCKD decreased from 213.45 ± 63.60 mg/dL to 129.13 ± 46.23 mg/dL (in CG from 210.57 ± 36.45 mg/dL to 206.44 ± 50.03 mg/dL) -HDL in LCKD increased from 36.71 ± 4.42 mg/dL to 52.99 ± 7.77 mg/dL (in CG from 44.14 ± 5.07 to 43.01 ± 5.03 mg/dL)	[50]
RCT, 2017	Comparison of the effects of a ketogenic diet vs. a moderate-carbohydrate diet on overweight adults with type 2 diabetes mellitus or pre-diabetes.	Very low-carbohydrate ketogenic diet (VLCKD) vs. moderate-carbohydrate, calorie-restricted, low-fat diet (MCCRD)	-In VLCKD, there was a significant reduction in TG from 102.6 mg/dL (81.8, 123.4) to 86.2 mg/dL (68.6, 103.7) in the 6th month and 92.7 mg/dL (73.6, 111.7) in the 12th month (in MCCRD from 158.9 mg/dL (128.8, 189.1) to 143.2 mg/dL (115.6, 170.9) in the 6th month and 173.4 mg/dL (138.1, 208.7) in the 12th month) -In VLCKD, there was an increase in HDL from 48.4 mg/dL (42.6, 54.2) to 51.9 mg/dL (45.7, 58.2) in the 6th month and 53.3 mg/dL (46.8, 59.8) in the 12th month (in MCCRD from 45.8 mg/dL (40.6, 51.0) to 48.1 mg/dL (42.5, 53.6) in the 6th month and 48.9 mg/dL (43.3, 54.5) in the 12th month) -In VLCKD, there was an increase in LDL from 88.7 mg/dL (76.3, 101.1) to 97.9 mg/dL (85.4, 110.5) in the 6th month and 95.6 mg/dL (82.3, 108.9) in the 12th month (in MCCRD from 98.1 mg/dL (86.4, 109.8) to 88.1 mg/dL (76.0, 100.1) in the 6th month and 96.1 mg/dL (83.7, 108.5) in the 12th month)	[51]
RCT, 2015	Evaluating the effects of ω-3 supplementation during a ketogenic diet in overweight subjects.	Ketogenic diet (KD) vs. ketogenic diet + ω-3 supplementation (KDO3)	In both dietary versions, there was a reduction in TC, LDL, TG and an increase in HDL. -TC in KD decreased from 217.25 ± 15.84 mg/dL to 201.28 ± 6.79 mg/dL (in KDO3 from 222.39 ± 6.10 mg/dL to 204.52 ± 9.78 mg/dL) -LDL in KD decreased from 133.41 ± 15.86 mg/dL to 123.60 ± 7.99 mg/dL (in KDO3 from 136.98 ± 7.06 mg/dL to 127.56 ± 7.19 mg/dL) -TG in KD decreased from 237.81 ± 20.26 mg/dL to 197.27 ± 6.1 mg/dL (in KDO3 from 230.79 ± 25.66 mg/dL to 185.54 ± 9.64 mg/dL) -HDL in KD increased slightly from 36.28 ± 2.23 mg/dL to 39.25 ± 1.37 mg/dL (in KDO3 from 39.55 ± 2.99 to 40.25 ± 2.63 mg/dL)	[52]
RCT, 2012	Comparison of the efficacy and metabolic impact of ketogenic and hypocaloric diets in obese children and adolescents.	Ketogenic diet (KD) vs. hypocaloric diet (HD)	-In KD, there was an increase in TC from 4.4 ± 0.85 mmol/L to 4.63 ± 0.75 mmol/L (in HD from 4.05 ± 0.94 mmol/L to 4.03 ± 0.89 mmol/L) -In KD, there was an increase in HDL from 1.27 ± 0.26 mmol/L to 1.38 ± 0.25 mmol/L (in HD from 1.13 ± 0.20 mmol/L to 1.23 ± 0.23 mmol/L) -In KD, there was an increase in LDL from 2.72 ± 0.69 mmol/L to 2.86 ± 0.65 mmol/L (in HD from 2.6 ± 0.83 mmol/L to 2.55 ± 0.77 mmol/L) -In KD, there was a reduction in TG from 0.83 ± 0.35 mmol/L to 0.81 ± 0.39 mmol/L (in HD from 0.89 ± 0.57 mmol/L to 0.80 ± 0.40 mmol/L)	[53]

**Table 2:** Effects of the ketogenic diet on blood pressure.

Type of Research, Year	Purpose of the Study	Diet Type	Blood Pressure Changes	References
Prospective pilot clinical trial, 2023	Evaluate the effect of very low-calorie ketogenic diet (VLCKD) on blood pressure (BP) in women with obesity and hypertension.	Very low-calorie ketogenic diet (VLCKD)	Relative to baseline values, after 45 days, there was: -a reduction in systolic blood pressure by an average of −12.89% (from an average of 140.88 ± 8.99 mmHg to 122.56 ± 10.08 mmHg) -a reduction in diastolic blood pressure by a mean of −10.77% (from a mean of 88.90 ± 6.71 mmHg to 78.94 ± 6.68 mmHg).	[206]
Prospective study, 2023	Evaluate the efficacy and safety of VLCKD on non-alcoholic fatty liver disease (NAFLD) and parameters commonly associated with this condition in overweight and obese subjects who did not take any drugs.	Very low-calorie ketogenic diet (VLCKD)	Relative to the initial values, after 8 weeks, there was: -a reduction in systolic blood pressure from an average of 133.51 ± 12.86 mmHg to 123.27 ± 10.51 mmHg -a reduction in diastolic blood pressure from a mean of 81.73 ± 8.09 mmHg to 75.27 ± 7.84 mmHg.	[207]
RCT, 2022	Assessment of the clinical advantage of combining two preoperative strategies (continuous positive airway pressure (CPAP) and low-calorie ketogenic diet (LCKD)) compared to CPAP alone, to improve apnea–hypopnea index (AHI) score, hypertension (HTN), dyslipidemia (DLP), insulin resistance (IR) and C-reactive protein (CRP) levels in patients with severe obesity and obstructive sleep apnea syndrome (OSAS) scheduled for bariatric surgery (BS).	Low-calorie ketogenic diet (LCKD) + continuous positive airway pressure (CPAP) vs. only continuous positive airway pressure (CPAP)	LCKD + CPAP vs. CPAP: -greater mean reduction in systolic blood pressure from 142.8 ± 13.3 mmHg to 133 ± 11.9 mmHg (in CPAP from 134.2 ± 10.4 mmHg to 130 ± 9.7 mmHg) -increased mean diastolic blood pressure reduction from 85.4 ± 8.38 mmHg to 78.7 ± 6.43 mmHg (on CPAP from 87 ± 11.6 mmHg to 82 ± 9.5 mmHg).	[45]
Pilot clinical trial, 2022	Investigate the efficacy of a very-low-carbohydrate ketogenic diet (VLCKD), known as Nic’s Ketogenic Diet, for 140 days on cardiometabolic markers in healthy adults with mildly elevated low-density lipoprotein cholesterol (LDL-C).	Very-low-carbohydrate ketogenic diet	-Systolic blood pressure decreased by 5.3% from baseline on day 140 of VLCKD. -There was a significant increase in diastolic blood pressure on day 28; however, there was no significant change on days 56, 70, 84, 112 and 140.	[211]
RCT, 2020	Comparison of the efficacy, safety and effect of 45-day isocaloric very-low-calorie ketogenic diets (VLCKDs) incorporating whey, vegetable or animal protein on the microbiota in patients with obesity and insulin resistance, to test the hypothesis that the protein source may modulate the response to VLCKD interventions.	Isocaloric VLCKD regimens (≤800 kcal/day) containing whey (WPG), plant (VPG) or animal protein (APG)	Relative to baseline values, after 45 days, there was: -a reduction in mean systolic pressure values (in WPG from 132 ± 10 mmHg to 124 ± 13 mmHg, in VPG from 131 ± 8 mmHg to 121 ± 10 mmHg, in APG from 129 ± 9 mmHg to 121 ± 16 mmHg) -a reduction in mean diastolic pressure values (on WPG from 78 ± 11 mmHg to 70 ± 9 mmHg, on VPG from 78 ± 10 mmHg to 72 ± 10 mmHg, on APG from 78 ± 10 mmHg to 71 ± 9 mmHg)	[49]
Meta-analysis, 2020	Evaluation of the efficacy and safety of VLCKD in overweight and obese patients.	Very-low-calorie ketogenic diet (VLCKD)	VLCKD was associated with an average reduction in systolic blood pressure of −8 mmHg and diastolic blood pressure of −7 mmHg.	[60]
RCT, 2017	Comparison of the effects of a ketogenic diet vs. a moderate-carbohydrate diet in overweight adults with type 2 diabetes mellitus or pre-diabetes.	Very-low-carbohydrate ketogenic diet (VLCKD) vs. moderate-carbohydrate, calorie-restricted, low-fat diet (MCCRD)	There was a slight reduction in diastolic blood pressure in both groups: -in LCK from an average of 77.1 mmHg (74.0, 80.3) to 77.1 mmHg (74.0, 80.1) in the 6th month and to 75.6 mmHg (72.5, 78.8) in the 12th month -in MCCRD from an average of 81.1 mmHg (78.2, 84.1) to 80.8 mmHg (77.9, 83.7) in the 6th month and 78.4 mmHg (75.5, 81.4) in the 12th month. There were small changes in systolic blood pressure in both groups: -in LCK from an average of 127.1 mmHg (121.9, 132.3) to 130.7 mmHg (125.7, 135.7) in the 6th month and 130.3 mmHg (125.2, 135.4) in the 12th month -in MCCRD from an average of 129.2 mmHg (124.6, 133.7) to 130.4 mmHg (125.6, 135.1) in the 6th month and 127.5 mmHg (122.7, 132.4) in the 12th month.	[51]
Systematic review with meta-analysis, 2013	Investigate whether individuals assigned to a VLCKD (i.e., a diet with no more than 50 g carbohydrates/d) achieve better long-term body weight and cardiovascular risk factor management when compared with individuals assigned to a conventional low-fat diet (LFD, i.e., a restricted-energy diet with less than 30% of energy from fat).	Very-low-carbohydrate ketogenic diet (VLCKD) vs. dieta niskotłuszczowa z deficytem kalorycznym (LFD)	-There was a significant difference in favor of the VLCKD in lowering diastolic blood pressure (WMD—1–43 (95% CI—2–49, 0–37) mmHg) -to a lesser extent, there was a difference in lowering systolic blood pressure (WMD in favor of the VLCKD—1–47 (95% CI—3–44, 0–50) mmHg).	[212]
RCT, 2012	To compare the efficacy and metabolic impact of ketogenic and hypocaloric diets in obese children and adolescents.	Ketogenic diet (KD) vs. hypocaloric diet (HD)	Mean systolic blood pressure decreased in KD from 110 ± 13 mmHg to 108 ± 13 mmHg, while diastolic blood pressure increased from a mean of 66 ± 10 mmHg to 68 ± 8 mmHg. In HD, there was a non-significant reduction in systolic blood pressure from 107 ± 9 mmHg to 106 ± 11 mmHg, and diastolic blood pressure from a mean of 65 ± 10 mmHg to 62 ± 11 mmHg.	[53]
RCT, 2010	Comparison of the effects of a low-carbohydrate ketogenic diet (LCKD) and orlistat therapy in combination with a low-fat diet (O + LFD) as a weight loss therapy on key parameters, i.e., body weight, blood pressure, fasting serum lipids and glycemic parameters.	Low-Carb Ketogenic Diet (LCKD) vs. low-fat diet in combination with orlistat (LFD + O)	Relative to baseline values after 48 weeks, there was a significantly greater reduction in blood pressure in the LCKD group compared to LFD + O: -average systolic blood pressure decreased by −5.94 mmHg (−1.5 mmHg in LFD + O) -average diastolic blood pressure decreased by −4.53 mmHg (in LFD + O by −0.43 mmHg).	[208]
RCT, 2010	To evaluate the effects of 2-year treatment with a low-carbohydrate or low-fat diet, each of which was combined with a comprehensive lifestyle modification program.	Low-carbohydrate diet vs. low-fat diet with a caloric deficit	There was a greater reduction in mean diastolic blood pressure in the low-carbohydrate group: -5.53 mmHg (−6.70 to −4.36) (vs. −3.05 mmHg (−4.29 to −1.81)) in the 3rd month; −5.15 mmHg (−6.49 to −3.82) (vs. −2.50 mmHg (−3.76 to −1.25)) in the 6th month; −3.25 mmHg (−4.74 to −1.76) (vs. −2.19 mmHg (−3.58 to −0.79)) in the 12th month; −3.19 mmHg (−4.66 to −1.73) (vs. −0.50 mmHg (−2.13 to 1.13)) in the 24th month. There was a slightly greater reduction in mean systolic blood pressure in the low-carbohydrate group: -7.74 mmHg (−9.59 to −5.89) (vs. −5.20 mmHg (−7.09 to −3.31)) in the 3rd month; −7.36 mmHg (−9.26 to −5.47) (vs. −6.97 mmHg (−8.89 to −5.05)) in the 6th month; −5. 64 mmHg (−7.62 to −3.67) (vs. −4.06 mmHg (−6.07 to −2.05)) in the 12th month; −2.68 mmHg (−5.08 to −0.27) (vs. −2.59 mmHg (−5.07 to −0.12)) in the 24th month.	[209]
RCT, 2003	Testing the hypothesis that severely obese subjects with a high prevalence of diabetes or metabolic syndrome would achieve greater weight loss, without detrimental effects on risk factors for atherosclerosis, while on a carbohydrate-restricted (low-carbohydrate) diet than on a calorie- and fat-restricted (low-fat) diet.	Low-carbohydrate diet (<30 g/d) vs. calorie- and fat-restricted diet	Relative to baseline values, after 6 months, there was: -a non-significant mean reduction in systolic blood pressure of 2 mmHg and diastolic blood pressure of 1 mmHg in the low-carbohydrate group -a non-significant mean reduction in systolic blood pressure of 2 mmHg and diastolic blood pressure of 2 mmHg in the low-carbohydrate and low-fat groups.	[210]

## Data Availability

Not applicable.
